# Evolution of host preference in anthropophilic mosquitoes

**DOI:** 10.1186/s12936-018-2407-1

**Published:** 2018-07-09

**Authors:** Chris Stone, Kevin Gross

**Affiliations:** 1 Illinois Natural History Survey, University of Illinois, 1816 S Oak St., Champaign, USA; 2 Department of Statistics, North Carolina State University, 2311 Stinson Dr., Raleigh, 27695 USA

**Keywords:** Host preference, Resource specialization, Adaptive dynamics, Mosquito, Insecticide-treated bed nets, Behavioural resistance, Mathematical model

## Abstract

**Background:**

Insecticide-treated bed nets (ITNs) have played a large role in reducing the burden of malaria. There is concern however regarding the potential of the mass distributions and use of ITNs to select for insecticide and behavioural resistance in mosquito populations. A key feature of the vectorial capacity of the major sub-Saharan African malaria vector *Anopheles gambiae* sensu stricto (s.s.) is its tendency to feed almost exclusively on humans. Here, an evolutionary model is used to investigate the potential for ITNs to select for increased zoophily in this highly anthropophilic species and how this is influenced by ecological and operational conditions.

**Results:**

The evolution of a single trait, namely the tendency to accept cattle as hosts, is modelled in mosquito populations which initially only bite humans. Thus, the conditions under which a resource specialist would broaden its diet and become a generalist are investigated. The results indicate that in the absence of insecticide-treated nets, host specialization in mosquitoes is either driven toward human specialization (when humans are more abundant than alternative hosts), or displays evolutionary bistability. The latter implies that the evolutionary endpoint relies on the initial trait value of the population. Bed nets select for increased zoophily while in use. When ITNs are removed, whether or not the population reverts to anthropophagic or zoophagic behaviour depends on whether the intervention had been maintained sufficiently long to drive the population past the evolutionarily unstable point.

**Conclusions:**

The use of ITNs is likely to select for an increase in the biting preference for cattle. Bed nets may thus alter the population composition of major vector species in a manner that has positive epidemiological ramifications. Whether populations are set on a trajectory toward increased zoophily following the cessation of intense bed net usage in an area depends on the composition of host communities as well as operational conditions. This has potential implications for bed net campaigns, particularly with an eye toward scaling down interventions following interruption of transmission. Further research on malaria mosquito feeding behaviour is warranted to explore the conditions under which such adaptive shifts may actually occur in the field.

## Background

The evolution of specialist or generalist habitat preferences and use has wide ramifications for the development and maintenance of species coexistence [1–4]. Because the availability of different resource or habitat types can shift along with species invasions, changes in species’ geographic ranges, and other types of human-mediated environmental changes such as deforestation or urbanization, species are likely to face changing selective pressures on their foraging behaviours. Thus, understanding the evolutionary dynamics of specialized resource usage under varying environments has implications for population management.

The existence of constraints or trade-offs is central to theoretical studies on the evolution of ecological specialization, whereby species that make use of a single resource do so more efficiently than would species that make use of two or more resources. Further, it has been shown that the shape and intensity of such trade-offs can determine evolutionary outcomes to a great extent [2, 3]. For instance, weak trade-offs have been shown to favour generalist strategies, while stronger trade-offs can lead to specialist genotypes. Other factors that have been shown to be influential include whether conditions fluctuate or are homogeneous, the scale (i.e., within particular habitats or over the entire system) at which density-dependent regulation operates, as well as the search time or abundance of resources [1, 5]. Neurological constraints related to signal processing efficiency have also been suggested to favour the evolution of specialized foraging behaviour [6, 7].

Despite the wide range of outcomes with regard to specialization in models, at least most phytophagous insects are highly host-specific [5]. In theoretical studies, trade-offs are posited on the logical grounds that in their absence an all-purpose generalist that performs optimally in all situations could evolve [3]. Evidence for trade-offs associated with resource use has however been ambiguous [8, 9], although this may in part be due the difficulty of capturing natural conditions in greenhouse or laboratory conditions [5]. Given the ongoing efforts to understand specialization, it is surprising that relatively few studies have focused on the evolution of specialization in other systems, such as in hematophagous insects [10]. Mosquitoes are of particular interest among this group as they introduce state-dependence, whereby density dependent competition for resources operates within the larval (aquatic) stages, while host choice and possible specialization relates to the blood-feeding behaviour on various vertebrate species by the adult female. In contrast to phytophagous insects, among mosquito species a diversity of degree of specialization is found, both between and within species [11]. For instance, while many mosquito species appear to be true generalists and are opportunistic in their blood-feeding behaviour, other species show strong and consistent preferences, either at the class level (e.g., a preference for mammals or birds) or at a species level (e.g., humans in the case of the anthropophilic vectors *Anopheles gambiae* and *Aedes aegypti*) 12–14].

Although host use by mosquitoes is, to an extent, plastic and affected by the relative abundance of various vertebrate species [15], host preferences likely do have a genetic basis. For instance, the hybrid ancestry of North-American *Culex pipiens* influences their preference for humans over birds [16], while a difference in a single odorant receptor gene has been linked to the anthropophilic biting behaviour of the domestic form of the yellow fever mosquito, *Aedes aegypti* [17]. Artificial selection experiments had previously illustrated that a strong preference to attack humans could shift to a preference for biting cattle in only a few generations in the malaria vector *An. gambiae* [18]. In a recent study on the generalist species *Anopheles arabiensis*, the genomes of individual cattle-fed and human-fed mosquitoes were sequenced and this revealed evidence for a genetic component for host choice. In particular, this study suggested that alleles related to the 3Ra chromosomal inversion may influence host preference in this species [19].

A thorough understanding of the proximate and ultimate causes of host specialization in mosquitoes remains elusive. Studies have been done on the strength of putative trade-offs related to feeding on the blood of different vertebrates and on the impact of host-defensive behaviour, in some cases showing a fitness advantage associated with feeding on a preferred host, while other cases suggest a trade-off may be either weak or non-existent [20–22].

The relative lack of insight into the genetic basis of host preference of mosquitoes, and how physiological, ecological and environmental factors interact to shape the selective pressures on host choice is surprising given that vector-borne disease transmission intensity is highly sensitive to variation in this trait [23]. It is also pertains to population management and vector control methods. This is perhaps most clearly the case for malaria, a major infectious disease of humans which continues to kill hundreds of thousands of people per year. A striking decline in prevalence and morbidity due to *Plasmodium falciparum* has occurred over the past decade, primarily due to the mass distribution of insecticide-treated bed nets (ITNs) in malaria endemic regions [24]. There is concern that the evolution of insecticide resistance may soon limit the efficacy of ITNs [25]. In addition to insecticide resistance mechanisms, mosquitoes have developed behavioural adaptations to bed nets. Such shifts in behaviour include changes in the peak biting time of the traditionally nocturnal anopheline vectors [26, 27], but changes in the proportion of blood meals that are taken on humans have also been described [28, 29]. While it is not always clear whether such shifts reflect a change in biting outcomes (which may result from changes in host availability) or in changes in biting preferences [30], it is certainly plausible that large-scale usage of bed nets among humans would change the selective pressures on mosquito host preference.

A simplified model of mosquito foraging and evolutionary dynamics of host preference in an anthropophilic mosquito, with access to only two different host types (e.g., cattle and humans), was developed. This stage-dependent model of mosquito population dynamics allowed for density-dependent regulation during the immature stage, with host-seeking decisions and egg-laying occurring during the adult stage. The objective was to explore the selective pressures on mosquito host preference and the adaptive responses that can be expected given different vertebrate host abundances, the strength of a trade-off, as well as the population coverage level of ITNs and the period of time for which these are deployed.

## Model description

### Population dynamics

Changes in the population size of immature and adult female mosquitoes are described by the following non-linear matrix model:

1 $$\begin{aligned} \begin{bmatrix} I_{(t+1)}\\ N_{(t+1)} \end{bmatrix} = \begin{bmatrix} e^{-(d_0 + d_1 I_{(t)})} e^{(-\delta )}&\epsilon \\ \frac{1}{2} e^{-(d_0 + d_1 I_{(t)})} (1-e^{(-\delta )})&p_d\end{bmatrix} \begin{bmatrix} I_{(t)}\\ N_{(t)} \end{bmatrix} \end{aligned}$$

For simplicity, the time delays associated with the egg and pupal stages (as well as pupal mortality) are ignored and all four larval instars are combined into a single stage. Further, adult male mosquitoes are not considered and females assumed to be able to reproduce immediately following emergence. Here $\epsilon$ represents the number of hatching eggs produced per day per female. Larvae die at a base rate determined by $d_0$, and a density-dependent rate determined by $d_1$. Immatures develop into adult mosquitoes at a rate $\delta$, which is assumed to be constant. A sex ratio of one half is assumed. Adult females survive each day with a probability $p_d$. Parameters and their values are described in Table 1.

**Table 1:** Description of parameters

Variable	Description	Value	Dim.
$\epsilon _{gc}$	Fecundity	50	Eggs per female per gonotrophic cycle
$\delta$	Immature development rate	0.1	d$^{-1}$
$d_{0}$	Immature mortality at low densities	0.1	d$^{-1}$
$d_{1}$	Additional mortality per conspecific	0.0005	d$^{-1}$
$\gamma _i$	Encounter rate with host type *i*	0.5	(0.1 d)$^{-1}$
$\sigma _i$	Probability of accepting host type *i*	Varies	–
$p_f$	Probability of surviving a single foraging bout	0.95	–
$\mu$	Max level of defensive mortality	0.5	–
$\omega$	Factor determining strength of trade-off	Varies	–

### Host choice

**Fig. 1:**
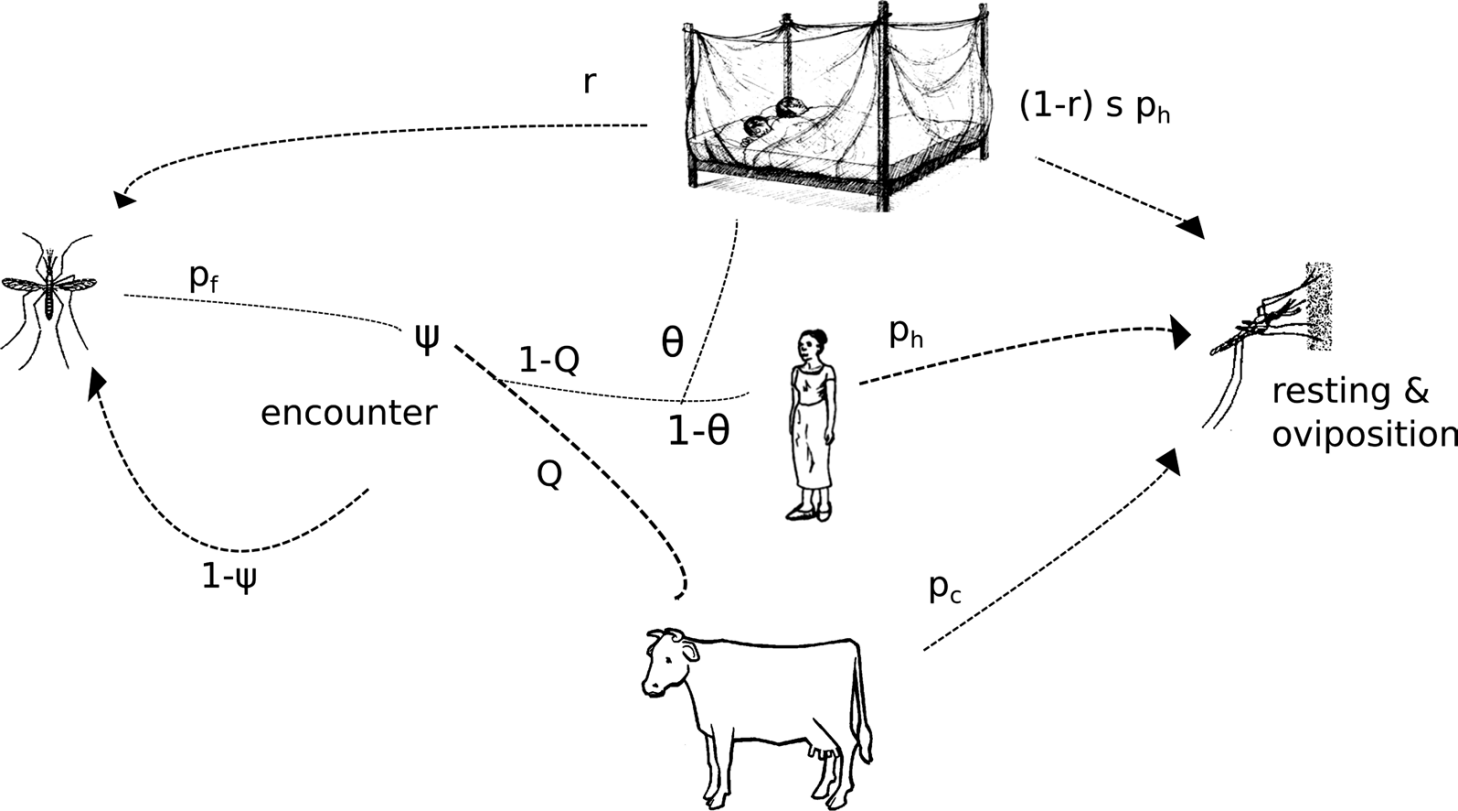
Diagram of the foraging process of malaria mosquitoes in the presence of two host types and insecticide-treated bed nets (The illustrations used in this diagram were adapted from [50])

The daily mortality and fertility rates of adult mosquitoes are derived from a cyclical foraging model (Fig. 1). Here, it is assumed that a single bout of host seeking is survived with probability $p_f$ (i.e., there is a base level of mortality associated with engaging in host-seeking flights) and that the probability of locating an acceptable host of any type during a single foraging bout is given by:

2 $$\begin{aligned} \psi = 1-e^{-(\sigma _h \gamma _h+ \sigma _c \gamma _c)} \end{aligned}$$

where $\gamma _h$ and $\gamma _c$ refer to the encounter rates of humans (*h*) and cattle (*c*). The innate preference for different species is indicated by $\sigma _h$ and $\sigma _c$, which are to be interpreted as the probability that a mosquito would attack a host if encountered. Successfully locating a host in a single foraging attempt occurs with probability $\psi p_f$, while failing to locate a host and surviving to make another attempt occurs with probability $(1-\psi ) p_f$. In total, surviving until a host is found occurs with probability:

3 $$\begin{aligned} p_{hs} = \frac{\psi p_f}{1-\left((1-\psi ) p_f\right)} \end{aligned}$$

Whether a female bites a host of a particular type *i* depends on both the host encounter rates ($\gamma _i$) and the probabilities of accepting different host types ($\sigma _i$). As the focus is on the highly anthropophilic *An. gambiae* s.s., the simplifying assumption is made that humans will always be accepted ($\sigma _h = 1$) and that the acceptance of cattle ($\sigma _c$) is the trait which can evolve. The objective is thus to explore under which conditions evolution is likely to drive an anthropophilic species towards a generalist strategy (though whether a population would in time start to avoid biting humans is not addressed). The conditional probability that a mosquito feeds on a cow, given that it locates a host, is given by:

4 $$\begin{aligned} Q = \frac{\sigma _c \gamma _c}{\gamma _h + \sigma _c \gamma _c} \end{aligned}$$

and the probability of feeding on humans is $1-Q$. Host-defensive behaviour ensures that not all mosquitoes that attempt to feed are successful and survive. It is assumed that this step is survived with probabilities $p_h$ and $p_c$, for biting humans and cattle, respectively. A mosquito survives foraging in the absence of ITNs with probability:

5 $$\begin{aligned} s_{f} = p_{hs} (Q p_c + (1-Q) p_h) \end{aligned}$$

### A trade-off between specialization and evasion of host defenses

A common assumption in studies on the evolution of host specialization is the existence of life history trade-offs which govern the effectiveness with which various resources can be exploited. By specializing on a particular host type, here it is assumed that a vector will improve its odds of evading that host type’s defensive behaviours, while at the same time, their vulnerability to other hosts could increase. To incorporate such a trade-off, $p_h$ and $p_c$ are written as functions of *Q*. The form for this trade-off is based on that of [3]. Thus, the performance (probability of surviving a feed on a given host) is based on the probability of attacking a particular host type:

6 $$\begin{aligned} p_{c{(Q)}} = 1 - \mu (1-Q)^\omega \end{aligned}$$

7 $$\begin{aligned} p_{h{(Q)}} = 1 - \mu Q^\omega \end{aligned}$$

Here $p_{h(Q)}$ and $p_{c(Q)}$ are the survival of a vector when feeding on a human or a cow, evaluated at the actual level of host use *Q*, $\mu$ is the maximum mortality due to host defensiveness, and $\omega$ determines the shape of the fitness function (i.e., how strong or weak the trade-off is).

### Gonotrophic cycle and daily survival probabilities

Following a successful blood meal, the remainder of the gonotrophic cycle has to be completed before another meal is taken. Possible supplementary blood meals taken within gonotrophic cycles (e.g., [31]) are not considered. This second phase of the cycle therefore encompasses resting, digestion of the blood meal, egg development and oviposition. Mosquitoes survive this phase with probability $s_r$. In total, surviving the gonotrophic cycle then occurs with probability:

8 $$\begin{aligned} p_{gc} = s_f s_r \end{aligned}$$

The approach of [32] is followed to translate between gonotrophic cycle and daily survival estimates. To do so, the durations of the foraging (first) and resting and oviposition (second) stages of the cycle have to be established. These durations are denoted with $\tau _1$ and $\tau _2$. For the duration of the foraging stage, $\tau _1$, there is an amount of time assumed to be associated with a typical foraging bout, $\tau _f$ (0.1 day). Then, $\tau _1 = \frac{\tau _f}{1-(1-\psi )p_f}$. For the duration of the resting stage, $\tau _2$, a fixed duration of 2.5 days is assumed. The biting rate, *f* is equal to $\frac{1}{\tau _1 + \tau _2}$:

9 $$\begin{aligned} f = 1 / \left(\frac{\tau _f}{1-(1-\psi )p_f} + \tau _2\right) \end{aligned}$$

Then daily survival is:

10 $$\begin{aligned} p_d = (s_f s_r)^f \end{aligned}$$

Daily fecundity is given by $\epsilon = \epsilon _{gc} f$, where $\epsilon _{gc}$ is the mean number of eggs produced per gonotrophic cycle.

### Foraging and survival in the presence of ITNs

Survival and fecundity of females are altered by ITN coverage (i.e., the proportion of humans sleeping under an ITN each night). This is both a function of mosquitoes being killed after contacting the insecticide on the net, or being diverted/repelled and having to spend additional time and effort locating a different host.

These effects are incorporated in the model as follows ([32, 33]). A mosquito can obtain a blood meal in a single foraging bout in the following ways: (1) by biting a non-human with probability $p_f \psi Q p_c$; (2) by biting an unprotected human: $p_f \psi (1-Q) (1-\theta ) p_h$, where $\theta$ is ITN coverage; (3) by biting a protected human: $p_f \psi (1-Q) \theta (1-r) s p_h$, where *r* is the probability that a mosquito encounters an ITN and is repelled, and *s* the probability that a mosquito is not killed by the insecticide and manages to feed on the human (e.g., finds it way through a hole in the net). In total, a mosquito is then successful with probability:

11 $$\begin{aligned} \kappa = p_f \psi (Q p_c + (1-Q) (1-\theta ) p_h + (1-Q) \theta (1-r) s p_h) \end{aligned}$$

A mosquito repeats or returns for another foraging bout with probability

12 $$\begin{aligned} \rho = (1-\psi ) p_f + \psi p_f (1-Q) \theta r. \end{aligned}$$

In total, a mosquito is then successful at obtaining blood with probability $\kappa + \kappa \rho + \kappa \rho ^2 + \kappa \rho ^3 + \ldots$, which is $\frac{\kappa }{1-\rho }$. The probability of surviving the host-seeking phase in the presence of ITNs is therefore:

13 $$\begin{aligned} s_n = \frac{p_f \psi (Q p_c + (1-Q) (1-\theta ) p_h + (1-Q) \theta (1-r) s p_h)}{1-((1-\psi ) p_f + \psi p_f (1-Q) \theta r)} \end{aligned}$$

In total, surviving the gonotrophic cycle occurs with probability $p_{gc} = s_n s_r$.

Estimates of daily mortality and fecundity in the presence of ITNs are derived from the feeding frequency, as before:

14 $$\begin{aligned} f(\theta ) = 1 / \left(\frac{\tau _f}{1-\rho } + \tau _2\right) \end{aligned}$$

### Analysis

An investigation of possible evolutionary trajectories is performed for a single trait, $\sigma _c$, the probability with which a mosquito will accept or attack cattle. The objective is to find out whether there are ecological conditions (i.e., changes in combinations of host encounter rates) or vector-borne disease interventions, specifically the use of ITNs, under which a host specialist would evolve toward a generalist strategy, and to what extent these outcomes depend on the strength of a trade-off between specialization and performance.

To do so, an adaptive dynamics approach is used to locate evolutionarily singular strategies [34, 35]. This entails evaluating the fitness of a resident population with a given trait value and locating for which value of that trait the population’s growth rate is equal to zero, while for any invading genotype the growth rate is negative. The appropriate measure of fitness or population growth for state-based, density-dependent, or stochastic models is the Lyapunov exponent, $\vartheta$ [36]. For populations with density-dependent growth rates which have a stable equilibrium (as the models used here do), $\vartheta$ is equal to the logarithm of the dominant eigenvector, $\lambda$, of the projection matrix of the invading genotype evaluated at the population equilibrium of the resident type [37, 38]. The evolutionary end points were graphically evaluated by using pairwise invasibility plots. These plots were created using an iterative, numerical method [39].

Additionally, the evolutionary process was simulated over time in order to investigate how the host preferences of mosquitoes would respond to large-scale roll-outs of ITNs at varying levels of population coverage. Because campaigns where long-lasting insecticidal bed nets are provided to large proportions of populations are typically performed with the intent to interrupt malaria transmission, there is the possibility that following successful malaria control such high levels of coverage will not be maintained indefinitely. It is therefore pertinent to also ask how the duration of such interventions affects the evolutionary dynamics of mosquito populations. These simulations were performed by iterating the population dynamics of mosquito populations that started off almost entirely anthropophilic (with values of $\sigma _c$ around 0.02). Offspring produced by females were assumed to either have inherited the trait value of the parent, or to have undergone a mutation and vary slightly. The number of mutated offspring were assumed to be binomially distributed based on a mutation probability of 0.1. Offspring that differed from their parent were than randomly assigned to a population with a trait value that was either slightly smaller or larger than the parent. The mutation step size was arbitrarily set to 0.002. The mutation rate and step size were chosen to reflect mutations that are relatively common, but of small effect.

## Results

**Fig. 2:**
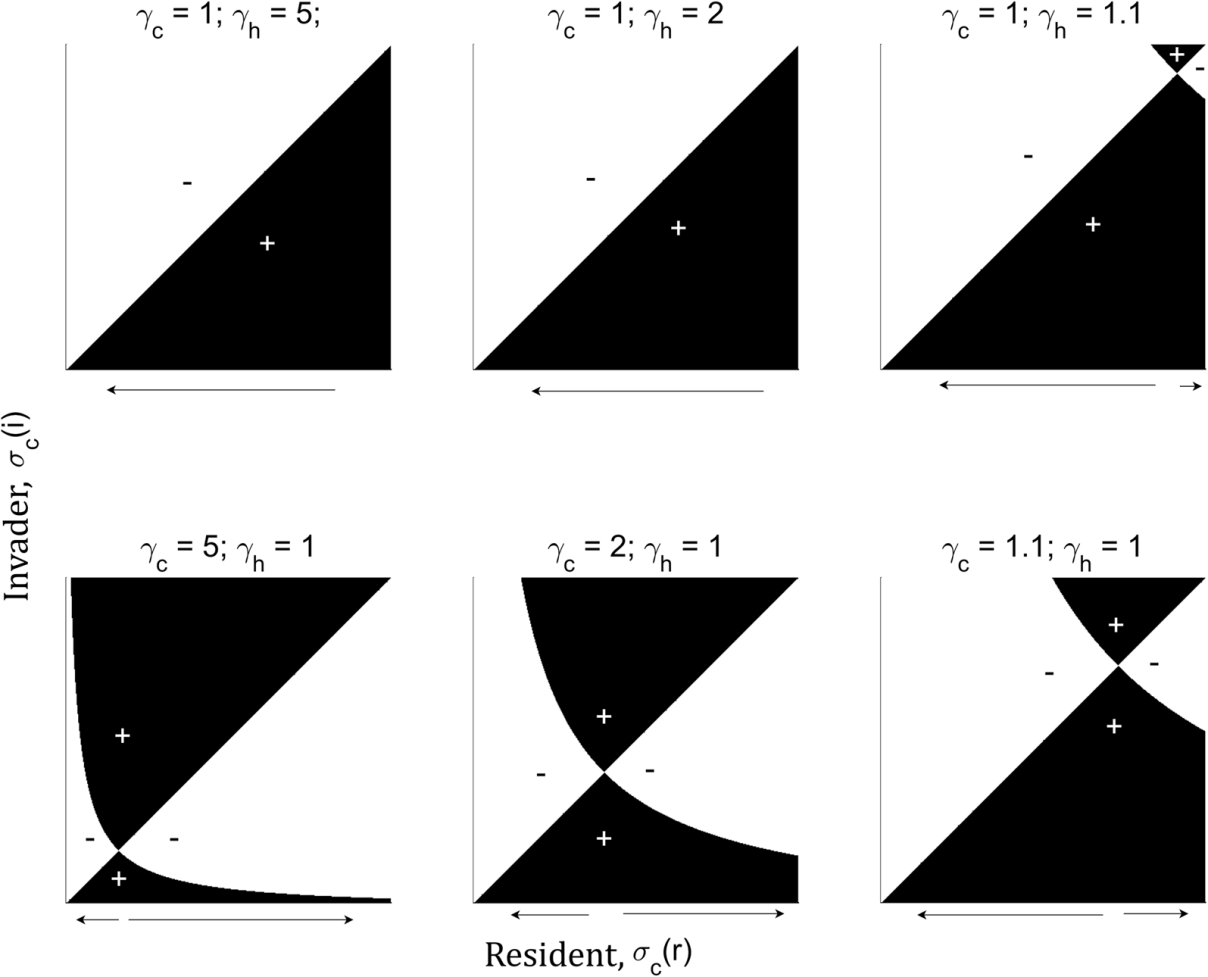
Pairwise invasibility plots for the host acceptability of cattle, $\sigma _c$, with values ranging from 0 to 1 for different levels of host type abundance or encounter rates ($\gamma _c, \gamma _h$), when humans are more common than cattle (top row) or vice versa (bottom row). The black sections indicate areas where invaders have a positive growth rate ($\vartheta > 0$) for a resident population with trait value $\sigma _c(r)$. The arrows below each panel indicate the direction in which host acceptability will evolve, depending on the initial value of $\sigma _c(r)$

Before investigating how the use of insecticide-treated bed nets may affect the evolution of host preference in malaria mosquitoes, it is useful to understand the evolutionary dynamics in the absence of human interventions. A specific question is whether there are ecological conditions under which a generalist strategy is likely to evolve in mosquitoes. Pairwise invasibility plots were created to understand the evolutionary end point of a sequential series of invasions by a rare mutant when the resident population has attained its equilibrium population size (Fig. 2). The outcomes suggest that in conditions where humans are significantly more abundant locally than are cattle (e.g., the first two panels of Fig. 2), the population will evolve toward complete anthropophily (i.e., $\sigma _c$ will evolve toward 0), regardless of the initial conditions of the population. Only when encounter rates are close to equal or favour cattle instead do the dynamics change. Specifically, an evolutionarily singular, unstable point appears. Thus, whether the population evolves towards specialization or generalization depends on the initial conditions of the mosquito population.

**Fig. 3:**
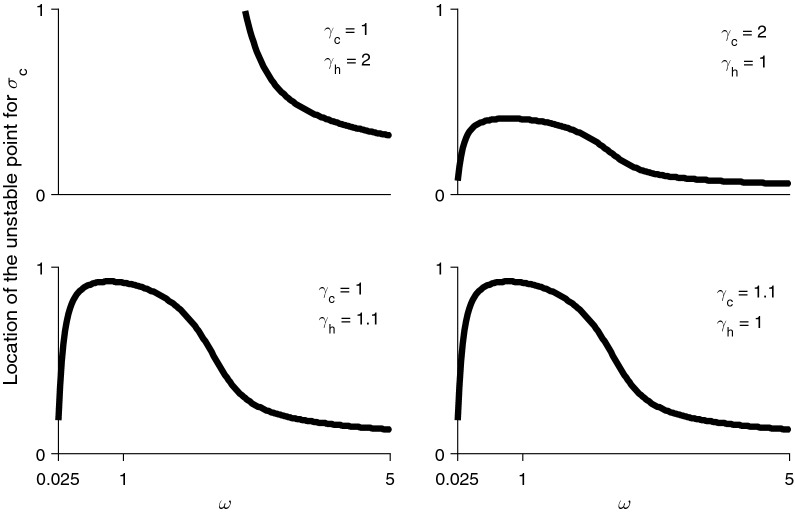
Unstable points for $\sigma _c$ at various combinations of encounter rates when the trade-off between performance and preference ranges from weak ($\omega > 1$) to strong ($\omega < 1$)

The evolutionary outcomes may also depend on the strength or the shape of trade-off function between performance on a particular resource and the extent to which mosquitoes specialize on those resources. To explore this, the ESS was numerically located as before and it was determined whether this point was stable (an attractor) or unstable (a repeller), along a range of values for $\omega$ (which determines the strength of the trade-off). The dynamics remained consistent with the insights derived from the pairwise invasibility plots. For instance, when humans are significantly more abundant, $\sigma _c$ evolves toward 0 or human specialization when the trade-off ranges from strong to moderately weak. It is only when the trade-off becomes very weak that an unstable point between values of 0 and 1 appears (Fig. 3). The location of this repeller does change and is affected by both the strength of the trade-off as well as by the composition of the host community. For instance, as $\omega$ becomes greater, the unstable point moves closer toward 0—indicating that a population with a wider range of starting conditions, or one which is perturbed by a relatively smaller amount from an anthropophilic starting condition, can evolve toward a generalist strategy. Additionally, the more abundant the alternative host type is, the greater the parameter space within which a generalist strategy is favoured.

**Fig. 4:**
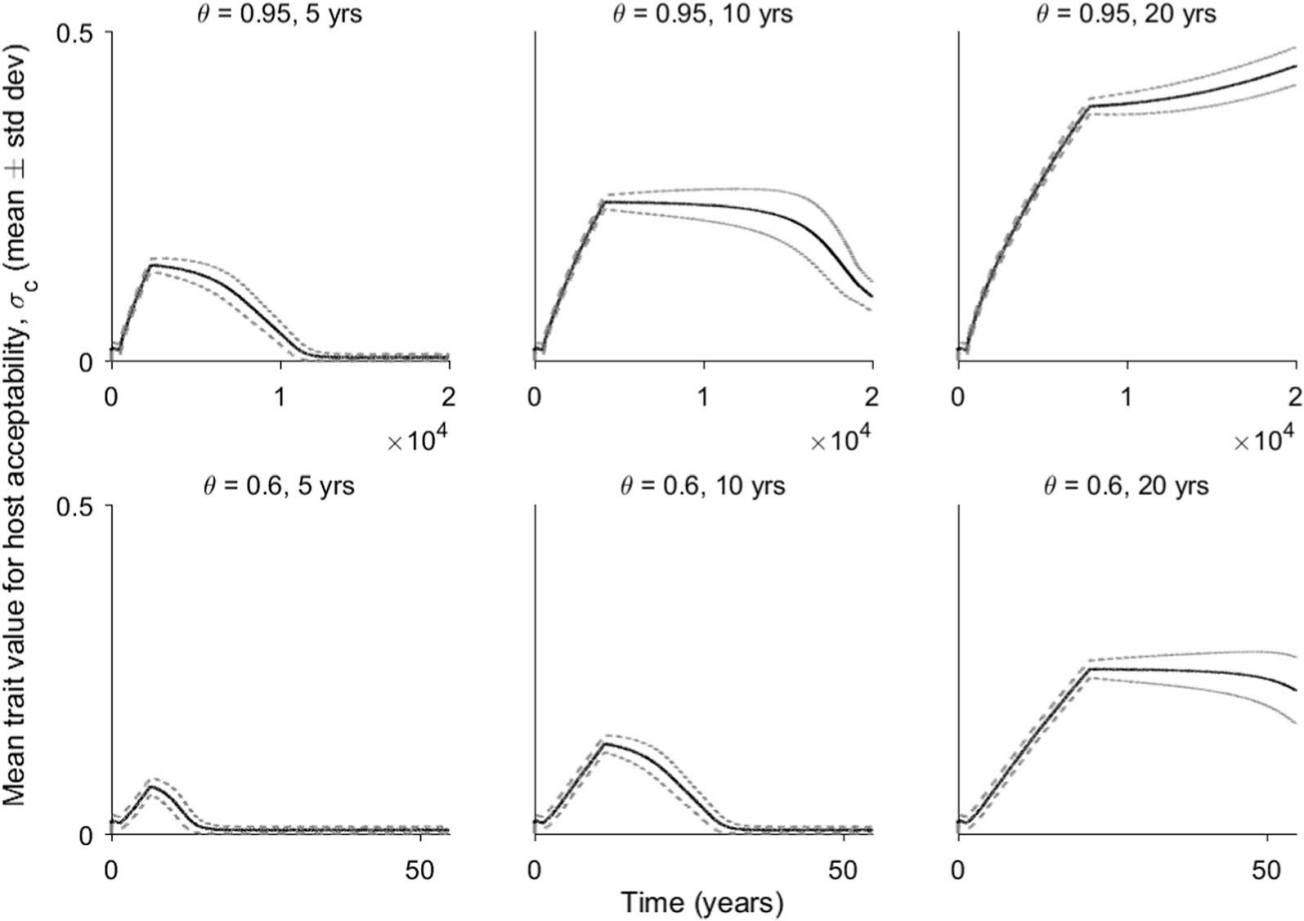
Evolution of $\sigma _c$ as simulated over time as bed nets are introduced, kept in place for a varying number of years at different levels of coverage ($\theta$), and following their withdrawal. In all these simulations the encounter rates for host types were 2 ($\gamma _c$) and 1 ($\gamma _h$), thus representing a situation were cattle are more abundant than humans

The simulations of the evolutionary process in the presence of ITNs highlight that this intervention modifies the fitness landscape of host acceptance drastically. Mosquito populations that start off biting humans almost exclusively will, over time, become more likely to also include cattle in their diet, as the mean trait value of $\sigma _c$ increases (Fig. 4). The evolutionary trajectory following withdrawal of the nets (e.g., if a mass distribution campaign has ended and nets are no longer replaced or maintained at scale) depends on both the level of population coverage, $\theta$, and the duration that the mosquito control measures are maintained. If net coverage is maintained at 95% for a relatively short period (e.g., 5 years), the population reverts to exclusive human biting over time. However, if ITN coverage is maintained at a high level for a longer period, the population is pushed beyond the unstable point and continues on a trajectory toward generalism even after removal of the ITNs.

**Fig. 5:**
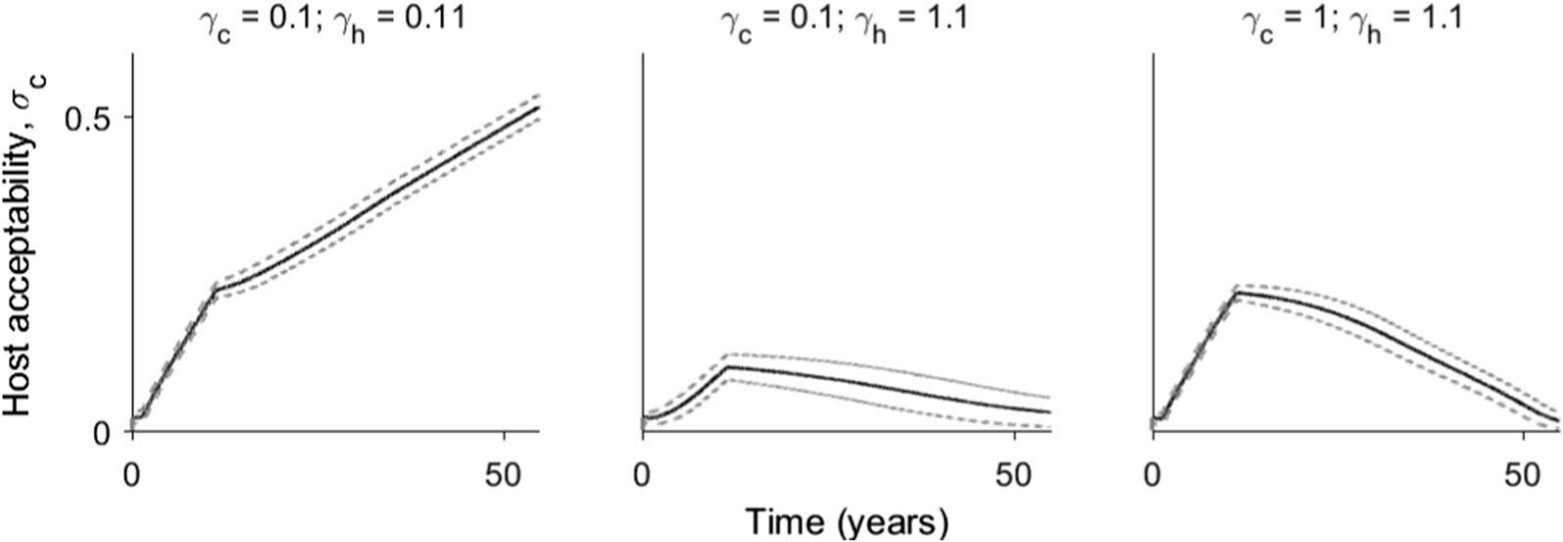
Evolution of $\sigma _c$ as simulated over time as bed nets are introduced, kept in place for ten years, and following their withdrawal. Coverage ($\theta$) was 95%. In these simulations the encounter rates for host types were varied, as indicated by the plot titles

It is not necessarily the case that such an outcome only occurs when cattle are more abundant than humans, although it does require a state of evolutionary bistability, i.e., the presence of an unstable point. When humans are only slightly more abundant, this outcome can also occur. Furthermore, the absolute values of encounter rates matter as well. For instance, under similar control parameters (10 years of 95% coverage) when the encounter rates favour humans by a similar proportion (i.e., values of $\gamma _c$ and $\gamma _h$ of 0.1 and 0.11, or 1 and 1.1, respectively), the evolutionary trajectory continues toward generalism when both hosts are rare, but reverts back to exclusive human feeding when encounter rates are higher (Fig. 5).

## Discussion

The main outcome of this study is the finding that the evolutionary outcomes of mosquito host specialization are either to never include cattle in their diet (exclusively biting humans) or to always bite cattle if given the opportunity (in which case they become generalists in our model). This outcome, where populations evolve toward one of two extreme options of specialization, has been described as a situation of evolutionary bistability [3]. Which specialization extreme is settled on in such cases depends on the initial trait values of the population, and particularly which side of a repelling point, separating the basins of attraction of these extremes, the population finds itself. The introduction of ITNs changes these dynamics by (temporarily) inducing directional selection toward increased zoophily, potentially setting the population on a trajectory toward generalism. It has to be stated that this is a general or strategic model with many inherent simplifications. Further ecological, physiological, and genetic details should be considered and studied before policy-level recommendations are made, although the current model results do highlight a number of considerations for malaria control or elimination programs.

First, the finding that the distribution and use of insecticide-treated bed nets will select for mosquito populations that are more likely to bite cattle has important epidemiological ramifications. This is because together with the biting frequency (the inverse of the duration of the gonotrophic cycle), the biting preference for humans of vectors is the parameter to which the basic reproduction number of malaria, $R_0$, is most sensitive, with relatively minor perturbations having larger effects as these parameters enter the equation of $R_0$ quadratically rather than linearly. It is recognized that ITNs affect *Plasmodium* spp. transmission through multiple modes, namely by increasing the mortality rate of *Anopheles* spp., by providing a barrier and increasing the duration of the feeding cycle, and potentially by diverting a proportion of bites to non-humans [32]. The current results suggest that during the course of an ITN program, the probability that a mosquito will feed on cattle may increase due to adaptive shifts in host preferences. As a result, the effect size of ITNs would be expected to increase with time. The most important result though is that such evolutionary shifts due to ITNs can in certain cases be permanent, and even set mosquito populations on an evolutionary trajectory toward increased zoophily, even after the use of bed nets has ceased or diminished. The latter is particularly relevant for ITNs as nets tend to degrade over time, either losing insecticidal efficacy as the nets are washed, or losing a barrier effect as holes of larger sizes increase with wear and tear [40]. In the absence of replenishments or redistribution of nets to communities, these interventions are therefore naturally time-limited. If there are continuing reductions in transmission pressure, this would be a considerable added benefit. Additionally, in certain areas bed nets may succeed in interrupting transmission and locally eliminating malaria. Given that maintaining nets indefinitely is costly, it could be tempting to cease distributions of bed nets at such a time (assuming there are other interventions in place to guard against resurgences following reintroductions). It is possible, however, that such decisions should not merely take into account epidemiological and economic concerns, but also evolutionary ones: it may in the long run pay to maintain ITN coverage long enough to ensure the population has been pushed beyond the evolutionary repeller and onto a trajectory toward zoophily.

As indicated previously, several assumptions were made in order to keep the model as simple as possible. For instance, the blood host community considered here consisted only of humans and cattle. While these two groups indeed do provide the majority of blood meals to *An. gambiae* s.s., other species are bitten occasionally as well (e.g., [41]). It is possible that in such a more diverse environment generalist strategies become more viable. In the absence of knowledge regarding putative correlations of species-specific preferences, or the shapes of fitness trade-offs in such more realistic and complex situations, it is hard to speculate and further work investigating the link between community diversity and specialization in vectors would clearly be useful.

Similarly, this study only allowed for the evolution of a single trait, the acceptability or attack probability on cattle ($\sigma _c$). In reality, it is possible that the attack rate on each distinct host type could evolve, so that rather than only moving between a specialization on one host type (here, humans) and a generalist strategy (where humans and cattle are both always attacked), specialization on the second host type could also evolve. It is possible then that the evolutionary endpoint identified here as a generalist strategy is not the true evolutionary endpoint, and *Anopheles* spp. may rather continue to evolve toward complete zoophily. This likely does not affect the main conclusion of this study. Adaptive dynamics models that allow for the evolution of multiple traits show that if the traits are independent (which we have assumed, in the absence of empirical evidence suggesting otherwise), they can be modelled independently [42]. If the traits covary (e.g., due to genetic linkage), the dynamics could possibly be more complicated. It does raise the question why mosquito species in general (i.e., with the exception of notable anthropophilic vectors such as *An. gambiae* or *Aedes aegypti*) often appear to be generalists. In other words, the ecological conditions that favour generalism in mosquitoes remain to be resolved. These conditions could include spatial or temporal variation in resources (whether in host quality, behaviour and dispersal, or relative abundance), where particularly temporal variability in hosts is thought to favour generalist strategies [43]. Another relevant complication which was not considered here relates to how vectors distribute themselves among hosts, and particularly whether they do so according to an ideal free distribution. In the current study, intraspecific competition was assumed to occur only in the larval stage, yet if there is competition at the level of blood-feeding as well, such an ideal free distribution could be important. There is indeed some evidence for increased defensiveness of hosts with increased densities of vectors [44]. It is also possible that humans would be more likely to use their bed nets or other control interventions under higher mosquito biting rates, although such behavioural change is perhaps more likely due to “nuisance” biting mosquitoes that occur at higher densities than malaria vectors typically do. Such refinements could be considered in a follow-up study., and could possibly lead to a broader range of parameters where a generalist strategy prevails.

A number of studies have now investigated the impact of the scaling-up of ITN usage on abundance, species composition, and blood-feeding behaviour of malaria mosquitoes. In certain locations, following the introduction of bed nets in areas where *An. gambiae* s.s. is present, this species has declined dramatically in abundance, often to near elimination, while more zoophilic or generalist (and closely-related) species, such as *An. arabiensis*, have persisted and proportionally become the dominant species and cause of residual malaria transmission [45–47]. The maintenance of anthropophilic behaviour is not limited to *An. gambiae* s.s. For instance, in Zambia, *An. arabiensis* displays anthropophilic tendencies and continued to do so for at least a two-year period after the introduction of insecticide-treated bed nets [48]. In such cases, these anophelines thus appear to maintain their anthropophilic biting habits, rather than shifting toward increased zoophily. The opposite has also been observed. In one case where ITNs were in use, *An. gambiae* s.s., while still predominantly feeding on humans, also included a variety of non-humans in its diet [41]. In another study, the human blood index of *An. gambiae* s.s. in an area where it had traditionally been strongly anthropophilic had dropped to only 53%, and a significant portion of those mosquitoes still feeding on humans had also taken a blood meal from a non-human animal [28]. An open question is why this discrepancy in outcomes is observed. The current study suggests that there can be evolutionary unstable points which can trap populations in specialist or generalist states. However, this is only the case in the absence of bed nets (e.g., when distribution campaigns are no longer sustained). While ITNs are in use, however, a shift toward zoophily is always expected in this model. A possible explanation for the discrepancy in outcomes may be that the decline in population size of *An. gambiae* s.s. can take place at a more rapid rate than evolutionary rescue [49] can occur. Speculatively, this could be affected by factors such as bed net coverage, the rate at which coverage is scaled-up, various socio-ecological factors, or the effective population size and amount of genetic variation present in the local mosquito populations. Likewise, this study did not consider competition with sympatric species, some of which (such as *An. arabiensis*) already possess more generalist host preference profiles. How or whether the presence of such other species affects the potential for evolutionary rescue is not clear. Additionally. an open question remains whether or how such adaptation would occur in the presence of insecticide resistance or the possibility of its evolution in the face of bed net coverage (e.g., [33]), or of other forms of behavioural resistance, such as adaptive shifts in the diel periodicity of blood-feeding (e.g., [27]). All of these would be interesting leads for follow-up studies, and would likely benefit from field studies which link population dynamics, genetics, and various behavioral changes within the same populations followed over time.

In conclusion, the mass distribution and use of insecticide-treated bed nets is likely to select for an increase in the acceptability or attack rate on cattle while ITNs are in use. This suggests that ITNs are not only highly effective control measures against the strongly anthropophilic vectors of malaria, they may temper and alter the population composition of such mosquito populations. Under certain conditions these selective pressures can enact permanent changes or set the population on a trajectory toward increased zoophily, even following the cessation of intense bed net usage in an area. The conditions under which this is true will depend on (and should therefore perhaps be considered in) operational design, as well as on ecological or social determinants. While further research on mosquito host feeding patterns under field conditions are warranted, studies that evaluate host utilization rates and potential shifts therein in areas where ITNs have been used for several years are particularly recommended.
